# Optimization of the ultrasonic roll extrusion process parameters based on the SPEA2SDE algorithm

**DOI:** 10.1038/s41598-022-07917-7

**Published:** 2022-03-09

**Authors:** Xiaoqiang Wang, Haojie Wang, Paigang Wang, Zhifei Liu

**Affiliations:** 1 School of Mechatronics Engineering, Henan University of Science and Technology, Luoyang, 471003 China; 2 Collaborative Innovation Center of Advanced Manufacturing of Mechanical Equipment, Luoyang, 471003 Henan China

**Keywords:** Mechanical engineering, Theory and computation

## Abstract

To obtain the optimal processing parameters of ultrasonic roll extrusion, 42CrMo bearing steel was taken as the research object, and the orthogonal test method was used to design an ultrasonic roll extrusion experiment with spindle speed, feed speed, static pressure and amplitude as parameters. Based on the orthogonal test data, the prediction models of surface roughness, surface residual stress and surface hardness were established by a multiple regression method, and the reliability of the model was verified. An algorithm combining SPEA2 and the shift density estimation strategy (SPEA2SDE) was introduced. The performance of the SPEA2SDE algorithm, NSGA II algorithm and SPEA2 algorithm is tested and compared on a three-dimensional test function set to verify its effectiveness. The SPEA2SDE algorithm are used to solve the multi-objective optimization model to obtain the optimal combination of processing parameters, and the ultrasonic roll extrusion experiment is carried out. The research results show that the surface roughness, surface residual stress and surface hardness optimized by the SPEA2SDE algorithm are in good agreement with the experimental values, and the average error is controlled within 10%, which shows that the algorithm can achieve high precision. It can effectively solve the multi-objective optimization problem of ultrasonic roll extrusion process parameters and can be used to guide actual production machining.

## Introduction

Ultrasonic roll extrusion processing is a new composite processing technology based on traditional rolling processing that uses the coupling effect of ultrasonic mechanical frequency vibration and static load rolling to treat the surface of metal materials. Compared with traditional surface strengthening technology, this method has the advantages of easy operation and high reliability and can significantly improve the surface properties of materials^1^. In recent years, ultrasonic roll extrusion technology has been widely used in the surface strengthening of metal materials and has become one of the hot spots in the manufacturing industry. In the process of ultrasonic roll extrusion, the combination of processing parameters has a great influence on the surface properties of materials, and the surface properties obtained by the optimal combination of processing parameters are also the best. The surface properties of metal materials are characterized by surface roughness, surface residual stress and hardness. To obtain the optimal surface properties of metal materials, multi-objective optimization of the surface roughness, surface residual stress and hardness is needed.

Junichiro Kumabe first introduced ultrasonic vibration turning into the field of machining, and then various forms of ultrasonic-assisted machining technology have become the focus of scholars at home and abroad. Liu et al.^2^ explored the principle of ultrasonic roll extrusion and verified the surface hardening index of a workpiece by experimental analysis and finite element simulation to analyse the influence of process parameters on the change in the evaluation index. Wang et al.^3^ studied the influence of ultrasonic rolling on the surface characteristics and fatigue properties of train axle steel and enhanced the fatigue properties of the material through ultrasonic impact to further ensure the safety and reliability of the train. Wang et al.^4^ analysed the influence of ultrasonic rolling extrusion process parameters on the surface roughness of bearing rings, predicted the surface roughness of bearing rings by the response surface method, and verified the accuracy of the model. Lai et al.^5^ studied the effect of ultrasonic surface rolling technology on the fatigue wear properties of 33Cr23Ni8Mn3N austenitic engine valve steel. The results showed that there were nanoscale layered grains on the surface of the treated material, which significantly improved the surface quality and physical properties of the material. Tan et al.^6^ studied the effect of ultrasonic surface rolling with different static pressures and feed speeds on the surface integrity of TC17 alloys and analysed the surface roughness, subsurface residual stress, microhardness and internal microstructure of the processed material. Zhao et al.^7^ carried out rotary ultrasonic rolling on a TC4 titanium alloy and analysed the influence of rolling pressure on the rolling depth and surface morphology. The results showed that ultrasonic rolling can significantly reduce the surface roughness, and the rolling pressure increased with increasing rolling depth. Li et al.^8^ studied the variation of surface characteristics of a TC4 titanium alloy with ultrasonic rolling force and established the theoretical model of ultrasonic rolling force and residual stress based on Hertz theory. ABAQUS software was used to simulate the effect of different ultrasonic rolling pressures on the residual stress.

Zhang et al.^9^ took a thin-walled ring sample as the research object, carried out a cutting test, established a regression model of the residual stress and deformation of the thin-walled ring workpiece, solved a multi-objective function by using a genetic algorithm, and obtained many groups of optimal processing parameter combinations, which can be used to guide actual production and processing. To study the multi-objective optimization problem of grinding face gear, Ming et al.^10^ used two methods to optimize the objective function and used the combination of the interior penalty function and genetic algorithm to improve the grinding efficiency and surface roughness. Devanathan et al.^11^ studied the influence of process parameters on the mechanical properties of composite materials in friction stir welding, designed the BBD method, designed an experiment, established a multi-objective optimization model for mechanical properties, and used the firefly algorithm to solve the multi-objective optimization of the two objective functions. Sara et al.^12^ used the sequential approximation optimization (SAO) of radial basis function (RBF) network to optimize the injection molding process parameters, which improved the product quality and productivity. The effectiveness of the method was verified by numerical analysis and experiments. Mia et al.^13^ used grey correlation analysis combined with the Taguchi method as the multi-objective optimization method to optimize the cutting force, surface roughness and cutting temperature of Ti-6Al-4V alloy turning. At the same time, the differences between single objective optimization and multi-objective optimization were compared and analysed.

In summary, many scholars at home and abroad have performed much research on ultrasonic roll extrusion technology and obtained better surface properties of metal materials. Some scholars have used different methods for the multi-objective optimization of process parameters, but they have not been applied to the multi-objective optimization of the surface properties of ultrasonic roll extrusion metal materials. Therefore, this paper takes 42CrMo material as the research object, designs an orthogonal experiment, and establishes a multi-objective optimization model of surface roughness, surface residual stress and hardness through a multiple regression method. An intelligent optimization algorithm is used to optimize the process parameters of ultrasonic roll extrusion, and the optimal control of the surface properties of metal materials is realized.

## Experiment of ultrasonic roll extrusion

### Experimental principle of ultrasonic roll extrusion

**Figure 1:**
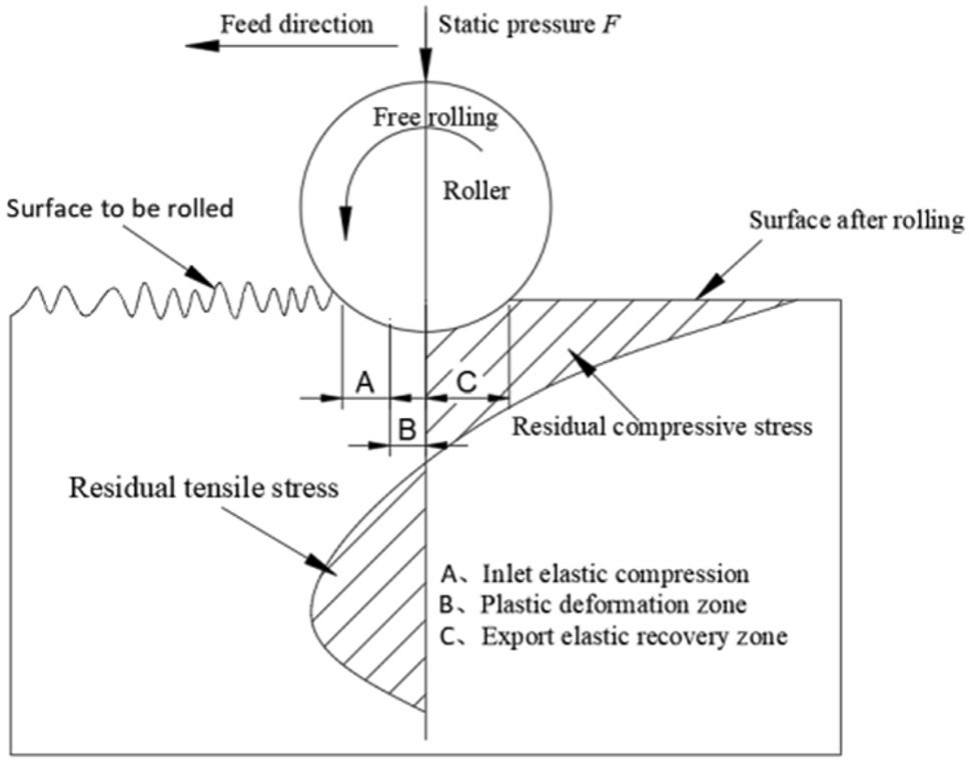
Principle diagram of ultrasonic rolling extrusion.

As shown in Fig. 1, a certain static pressure is applied to the roller when the sample is processed by ultrasonic rolling extrusion. The roller moves axially in the direction of feed. Under high-frequency vibration, the surface layer of the sample produces elastic–plastic deformation, and the metal flow on the surface layer is intense. The convex part of ultrasonic rolling extrusion is flattened and filled into the concave part, realizing micro "peak cutting and valley filling". The surface properties of the sample are improved significantly. When the roller processes the surface of the sample, volume compression occurs along the depth of the layer, resulting in plastic deformation. The uneven elastic–plastic deformation in Section B gradually begins to recover to Section C, but the plastic deformation layer on the surface hinders its recovery, resulting in a residual compressive stress on the surface.

### Test materials

42CrMo steel with a length of 320 mm and a diameter of 50 mm is selected for the ultrasonic roll extrusion test. The main chemical composition of the workpiece is shown in Table 1.

**Table 1:** Chemical composition of 42CrMo steel.

Category	Element name						
	C	Mn	Cr	Mo	Si	Ni	Fe
42CrMo	0.37%	0.77%	0.98%	0.21%	0.15%	0.04%	97.44%

### Test equipment

**Figure 2:**
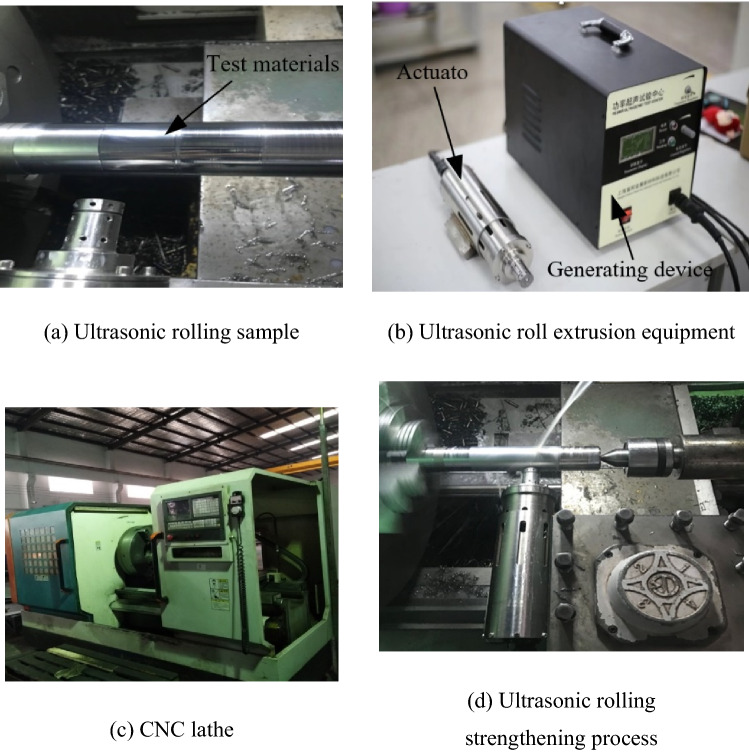
Ultrasonic roll extrusion.

The workpiece after ultrasonic rolling extrusion is shown in Fig. 2a. The ultrasonic rolling extrusion equipment is composed of an ultrasonic generating device and executive device, as shown in Fig. 2b. The ultrasonic rolling extrusion test is carried out on a CNC machine tool, as shown in Fig. 2c. Using ultrasonic rolling extrusion to process a bearing ring material is shown in Fig. 2d. The residual stress after strengthening was measured by an Xstress3000 X-ray instrument, the surface roughness of the machined bar was measured by a Time 3230 roughness shape measuring instrument, and the surface hardness was measured by an HVS-1000a microhardness measuring instrument.

### Orthogonal experimental design of ultrasonic roll extrusion

An orthogonal experimental design is adopted for ultrasonic roll extrusion processing. The processing parameters are workpiece speed n, feed speed F, amplitude A and static pressure F. A four factor and five level design L25(5^4^) is selected for the experiment. The factor level setting table is shown in Table 2, and the orthogonal experimental results are shown in Table 3.

**Table 2:** Factor level setting table.

Level	Process parameters of ultrasonic roll extrusion			
	Speed (A)	Feed rate (B)	Amplitude (C)	Static pressure (D)
	*n*/(r/min)	*f*/(mm/min)	*A*/μm	*F*/N
1	150	15	5	200
2	270	26	11	280
3	390	37	17	420
4	510	48	23	510
5	630	59	29	600

**Table 3:** Orthogonal test results.

Test number	Horizontal combination	$R_{a}$(µm)	$\sigma$(MPa)	Hardness HV
1	$A_{1} B_{1} C_{1} D_{1}$	0.635	− 886	641
2	$A_{1} B_{2} C_{2} D_{2}$	0.581	− 987	659
3	$A_{1} B_{3} C_{3} D_{3}$	0.370	− 1112	707
4	$A_{1} B_{4} C_{4} D_{4}$	0.338	− 1137	692
5	$A_{1} B_{5} C_{5} D_{5}$	0.302	− 1074	680
6	$A_{2} B_{1} C_{2} D_{3}$	0.523	− 1029	713
7	$A_{2} B_{2} C_{3} D_{4}$	0.483	− 1171	722
8	$A_{2} B_{3} C_{4} D_{5}$	0.381	− 1195	726
9	$A_{2} B_{4} C_{5} D_{1}$	0.646	− 919	631
10	$A_{2} B_{5} C_{1} D_{2}$	0.461	− 1077	663
11	$A_{3} B_{1} C_{3} D_{5}$	0.441	− 1175	748
12	$A_{3} B_{2} C_{4} D_{1}$	0.603	− 978	639
13	$A_{3} B_{3} C_{5} D_{2}$	0.569	− 996	669
14	$A_{3} B_{4} C_{1} D_{3}$	0.555	− 1029	720
15	$A_{3} B_{5} C_{2} D_{4}$	0.460	− 1176	738
16	$A_{4} B_{1} C_{4} D_{2}$	0.556	− 1038	691
17	$A_{4} B_{2} C_{5} D_{3}$	0.431	− 1168	741
18	$A_{4} B_{3} C_{1} D_{4}$	0.476	− 1011	753
19	$A_{4} B_{4} C_{2} D_{5}$	0.434	− 1139	757
20	$A_{4} B_{5} C_{3} D_{1}$	0.583	− 921	647
21	$A_{5} B_{1} C_{5} D_{4}$	0.434	− 1208	768
22	$A_{5} B_{2} C_{1} D_{5}$	0.445	− 998	772
23	$A_{5} B_{3} C_{2} D_{1}$	0.641	− 863	657
24	$A_{5} B_{4} C_{3} D_{2}$	0.478	− 914	683
25	$A_{5} B_{5} C_{4} D_{3}$	0.416	− 1056	719

## Establishment of the surface performance prediction model

Due to the complexity of the ultrasonic roll extrusion process, it is difficult to obtain an accurate mathematical expression of the surface performance. It is necessary to establish a mathematical model of surface performance by effective methods, and the quality of a model will directly affect the subsequent multi-objective optimization accuracy. Therefore, it is of great significance to establish a mathematical model with a high correlation between process parameters and surface properties for the optimization of ultrasonic roll extrusion process parameters.

### Performance modeling of ultrasonic rolling extrusion surface based on BP neural network

#### Principle of BP neural network

BP neural network is one of the most widely used neural networks in modeling. It has very strong self-learning ability and self-organizing learning ability. The neural network is used to establish the surface roughness prediction model of ultrasonic rolling extrusion.

**Figure 3:**
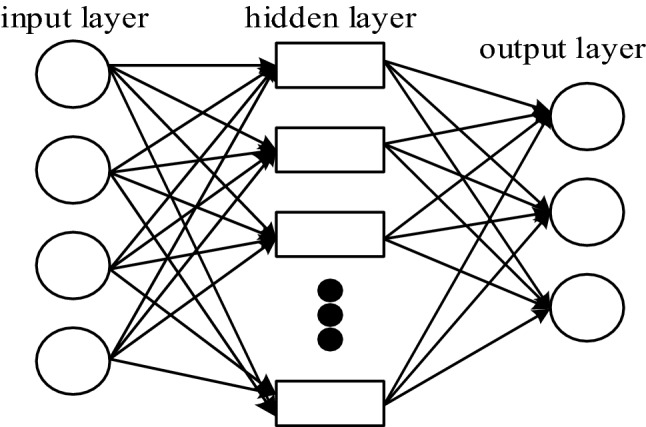
BP neural network topology.

In order to achieve reasonable control of surface properties, it is crucial to establish a predictive model of the relationship between surface properties and various processing parameters. While designing the BP neural network, several factors that affect the prediction accuracy need to be considered, such as the number of training samples, the number of neurons in the hidden layer, the transfer function and the learning algorithm. The topology of the ultrasonic rolling extrusion network is shown in Fig. 3. In the figure, there are 4 input layers (workpiece speed, feed rate, amplitude, static pressure), and 3 neurons in the output layer (surface roughness, surface residual stress, surface hardness).

#### Determining the number of hidden layers of neural networks

The number of neurons in the hidden layer is one of the main factors affecting the prediction accuracy, so the selection of the number of neurons in the hidden layer is a crucial step. The number of neurons in the hidden layer should be determined according to the designer's experience and repeated training, and the number of nodes in the hidden layer should be determined according to the empirical formula (1).

1 $$ u = \sqrt {x + n} + y $$

In the formula, *x* is the number of neurons in the input layer, *n* is the number of neurons in the output layer, and *y* is a constant within 1–10.

In order to make the prediction more accurate, the accuracy of the number of neurons in the hidden layer is compared, and the number of neurons in the hidden layer is calculated by formula (1) to be 3–12, and the number of neurons in the hidden layer is calculated by MATLAB. Experiments are carried out from 3 to 12, and the judgment is based on the sum of the percentage of the error of each layer in the output data, and the smallest is the best hidden layer. In this paper, the BP neural network is trained 100 times, and the average of the sum of the error percentages is obtained, so that the number of neurons in the hidden layer is more credible. The training function selects the Trainlm function, uses the above method to train the test data, and records the average of the sum of the error percentages of the neurons in each hidden layer.

**Figure 4:**
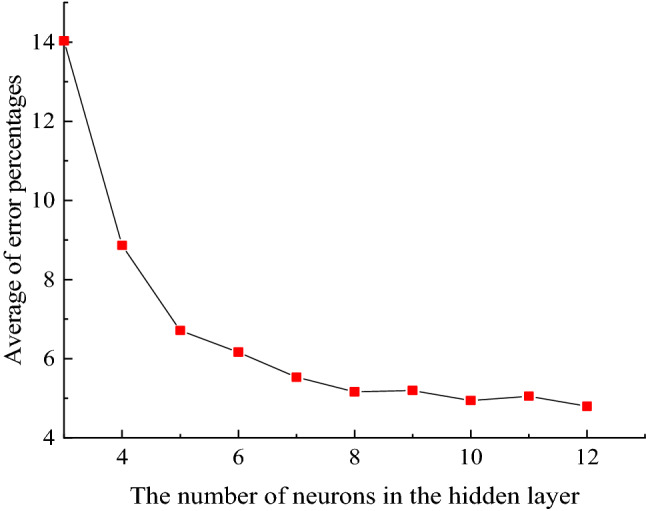
Comparison of the sum of the percentage of error and the average.

It can be found that after 100 training cycles from Fig. 4, when the number of neurons in the hidden layer is 12, the average sum of error percentages is the smallest, indicating that the model has the smallest prediction error when the number of neurons is 12. Stability is the best.

#### Neural network learning function selection

In the training of BP neural network, different learning functions will affect the learning rate, which will affect the accuracy of prediction. Therefore, the selection of transfer function and learning function is particularly important when training the target. In this paper, the hidden layer neuron transfer function tansig is used, the output layer uses logsig. The learning rate, the number of iterations, and the allowable error values are set as: 0.1, 2000, and 0.001, respectively. Different learning functions are used to train the orthogonal test results. Because the Trainlm function has the smallest error when training the surface performance, the learning function is selected as Trainlm.

#### BP neural network simulation

In order to make the prediction more credible, another 5 sets of experiments were done to verify the surface performance prediction model. The results are shown in Table 4.

**Table 4:** Ultrasonic roll extrusion test results.

Serial number	*n*	*f*	*F*	*A*	$R_{a}$	$\sigma$	$HV$
1	300	20	350	13	0.584	− 982	− 672
2	320	23	280	15	0.512	− 1078	− 702
3	400	21	300	14	0.523	− 975	− 679
4	430	25	460	20	0.471	− 1045	− 705
5	500	30	520	17	0.397	− 1066	− 711

If it is not convincing to directly use 25 sets of orthogonal tests for training to predict surface performance, Table 3 data are selected for neural network training, and Table 4 is used as test data to verify the training accuracy. After BP neural network training, the predicted value is simulated, and the comparison between the predicted result and the test sample is shown in Table 5.

**Table 5:** Test and predicted values of the surface performance of BP neural network model.

$R_{a}$/μm			$\sigma$/MPa			Hardness/HV		
Test value	Predicted value	Error (%)	Test value	Predicted value	Error (%)	Test value	Predicted value	Error (%)
0.584	0.654	11.9	− 982	− 1130	15.0	672	736	9.5
0.512	0.472	7.8	− 1078	− 1175	8.9	702	751	6.9
0.523	0.583	11.4	− 975	− 1064	9.1	679	723	6.4
0.471	0.495	5.0	− 1045	− 1152	10.2	705	655	7.1
0.397	0.472	18.8	− 1066	− 902	15.3	711	763	7.3

It can be seen from Table 5 that the maximum prediction error percentage of surface roughness is 18.8%, the maximum prediction error percentage of surface residual stress is 15%, and the maximum error percentage of surface hardness is 9.5%. The errors of surface roughness and surface hardness are more than 10%, which means that the BP neural network is used to predict the surface performance is inaccurate and the error is large. This is because the BP neural network is easy to fall into the local optimum during training, and the performance of the ultrasonic rolling extrusion surface cannot be predicted more accurately. A good prediction model, the following uses the RBF neural network to establish a prediction model for the performance of the surface layer.

### Surface performance modeling of ultrasonic rolling extrusion based on RBF neural network

The radial basis (RBF) neural network is composed of a three-layer feedforward neural network with a single hidden layer, in which the input layer nodes only play the role of transmitting signals, and the kernel function of the hidden layer nodes adjusts the parameters, and the input layer is generated locally. In response, the hidden layer adopts a nonlinear function, and the output layer node usually adopts a linear function. Different from other neural network structures, the function function of the hidden layer is a Gaussian function, and other neural networks have the characteristics of global response, and this kind of network has the ability of local approximation. Local optimum problem. In order to study the relationship between the process parameters of the ultrasonic rolling bearing ring and the performance of the surface layer, the number of neurons in the input layer is set to 4 (workpiece speed, feed speed, amplitude, static pressure), and the number of neurons in the output layer is 3 (surface roughness, residual stress, hardness).

#### RBF neural network structure design

In the establishment of the surface layer performance prediction model, the number of neurons in the hidden layer is the main factor affecting the prediction accuracy. In order to make the prediction performance better, the RBF neural network is trained, and different SPREAD (expansion coefficient of radial basis function) is selected. Through network training, each predicted value of the surface layer performance and the number of training times are obtained, and the appropriate number of neurons in the hidden layer is finally determined, and MATLAB is used to perform cyclic training on the radial basis network. The mean square error is set to 0.001, MN (neural The maximum number of cells) is 30, DF (the number of neurons added in each training) is the default value of 1, and the mean square error of different spread values is compared, and finally the number of neurons in the hidden layer is determined.

Use the above method to train the orthogonal test data, and record each SPREAD value, training times and mean square error. It is found from Table 6 that when the spread value is 1, the mean square error is the smallest, indicating that when the model spread value is 1, the mean square error is the smallest. The prediction error is the smallest and the accuracy is the highest. At this time, the number of training times is 24. Therefore, the number of neurons in the hidden layer of the RBF neural network model is 24, and the spread value is 1, so the RBF neural network structure is 4-24-3.

**Table 6:** Training situation of different radial basis function expansion coefficients.

Spread	1	3	5	7	10	13	16	19
Mean squared error	5.4E−28	1.1E−19	5.3E−17	5.8E−15	5.8E−15	6.9E−13	3.2E−10	5.28E−11
Training times	24	25	24	24	25	24	24	24

#### RBF neural network prediction results and analysis

The ultrasonic rolling and squeezing orthogonal test results are brought into the RBF neural network for training. When the number of training steps reaches 24, the training accuracy is the highest. In order to make the trained model more credible, the test data is brought into the trained model. The actual and predicted values and errors of the surface performance are shown in Table 7.

**Table 7:** Test and predicted values of the surface performance of RBF neural network model.

$R_{a}$/μm			$\sigma$/MPa			Hardness/HV		
Test value	Predicted value	Error (%)	Test value	Predicted value	Error (%)	Test value	Predicted value	Error (%)
0.584	0.551	5.6	− 982	− 1060	7.9	672	695	3.4
0.512	0.465	9.17	− 1078	− 1168	8.3	702	663	5.5
0.523	0.492	5.9	− 975	− 1010	3.5	679	699	2.9
0.471	0.448	4.8	− 1045	− 1115	6.7	705	738	4.6
0.397	0.362	8.8	− 1066	− 1125	5.8	711	749	5.3

It can be seen from Table 7 that the average error percentages between the actual and predicted values of surface roughness, residual stress and hardness are: 6.85%, 6.44% and 4.34%. In the prediction of surface roughness, residual stress and hardness, the second group of data has the largest error percentage, which is 9.17%, 8.3% and 5.5%. Overall, each predicted value is controlled within 9%. RBF neural network It is much higher than the prediction accuracy of BP neural network.

### Surface performance modelling by the multiple regression method

#### Multiple regression modeling process

Taking workpiece speed n, feed speed f, amplitude A and static pressure F as independent variables, the non-linear functional relationship between surface properties and ultrasonic roll extrusion process parameters is established. The empirical model between process parameters and surface properties is established in exponential form^14^.

2 $$ R_{a} ,\;\sigma ,\;HV = C \times n^{{b_{1} }} \times f^{{b_{2} }} \times F^{{b_{3} }} \times A^{{b_{4} }} $$

where C is a constant term and *b*_*1*_, *b*_*2*_, *b*_*3*_, and *b*_*4*_ are the indexes of workpiece speed, feed speed, static pressure and amplitude, respectively.

To facilitate calculation, Eq. (2) is linearized, and the logarithm is taken on both sides of the equation:

3 $$ \log R_{a} ,\;\log \sigma ,\;\log HV = \log C + b_{1} \log n + b_{2} \log f + b_{3} \log F + b_{4} \log A $$

Let $y = \log R_{a} ,\;\log \sigma ,\;\log HV$, $k_{0} = \log C$, $k_{1} = \log n$, $k_{2} = \log f$, $k_{3} = \log F$, and $k_{4} = \log A$. Then, formula (3) can be transformed into a multiple linear regression equation as follows:

4 $$ y = k_{0} + k_{1} b_{1} + k_{2} b_{2} + k_{3} b_{3} + k_{4} b_{4} $$

The logarithmic transformation of each process parameter and surface roughness, residual stress and surface hardness in the results of the orthogonal test were carried out, and the surface roughness, residual stress and surface hardness were analysed by multiple linear regression using Minitab software respectively. The mathematical model of surface roughness, residual stress and surface hardness were established, as shown in Eqs. (5), (6) and (7).

5 $$ R_{a} = 10^{0.717} n^{0.0864} f^{ - 0.1049} A^{ - 0.0792} F^{ - 0.3902} $$

6 $$ \sigma { = } - 10^{2.525} n^{ - 0.0087} f^{ - 0.0147} A^{0.0412} F^{0.1902} $$

7 $$ HV = 10^{2.4366} n^{0.04618} f^{ - 0.02214} A^{ - 0.01136} F^{0.13133} $$

#### Model analysis

To judge the reliability and stability of the multiple regression mathematical model of surface roughness, surface residual stress and surface hardness in ultrasonic roll extrusion, variance analysis of the regression model was carried out. In ANOVA, the P value represents the credibility of the model. In many research fields, P < 0.05 is considered an acceptable error level. The smaller the p value is, the higher the credibility of the model is. Through the analysis of variance of Eqs. (4), (5) and (6), this shows that the F value of the surface roughness multiple regression model is 20.52 and P < 0.0001, which indicates that the model has reached a significant level. The F value of the surface residual stress multiple regression model is 12.63 and P < 0.0001, and the F value of the surface hardness multiple regression model is 86.7 and P < 0.0001. This shows that the multiple regression model can better reflect the correlation between the model and the experimental value.

#### Multiple regression method prediction results and analysis

Bring the test data in Table 4 into formula (5), formula (6), and formula (7), and obtain the predicted value of multiple regression. The experimental value and predicted value of ultrasonic rolling extrusion processing surface performance are shown in Table 8. As shown, the error of using the multiple regression method to establish the surface properties of ultrasonic rolling extrusion is small, and the average error percentages of surface roughness, surface residual stress and hardness are 5.18%, 5.08% and 3.38%, respectively. The mathematical model of rolling extrusion surface performance has good precision and small error.

**Table 8:** Test and predicted values of the surface performance of the multiple regression model.

$R_{a}$/μm			$\sigma$/MPa			Hardness/HV		
Test value	Predicted value	Error (%)	Test value	Predicted value	Error (%)	Test value	Predicted value	Error (%)
0.584	0.57	2.3	− 982	− 1033	5.1	672	698	3.8
0.512	0.54	5.4	− 1078	− 993	7.8	702	676	3.7
0.523	0.55	5.1	− 975	− 1003	2.8	679	691	1.7
0.471	0.452	3.8	− 1045	− 1100	5.2	705	728	3.2
0.397	0.434	9.3	− 1066	− 1114	4.5	711	743	4.5

### Comparative analysis of surface performance prediction models

**Figure 5:**
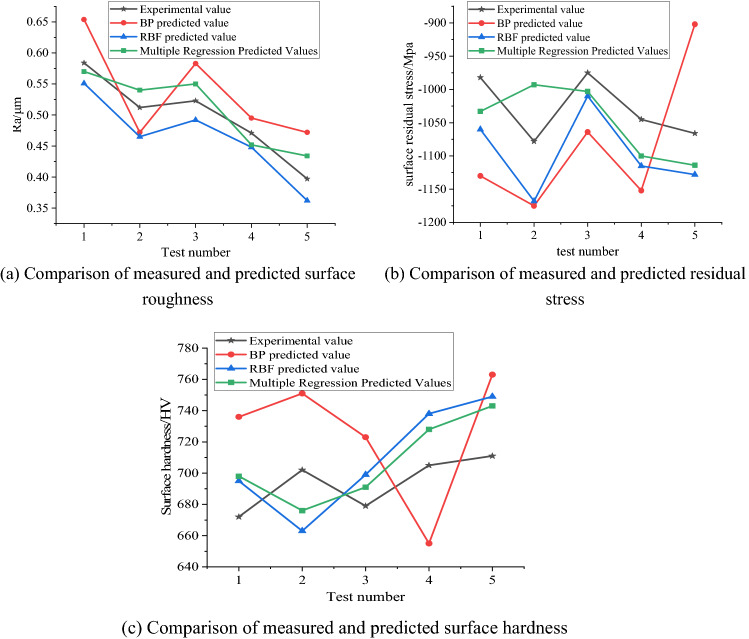
Comparison of measured and predicted surface performance.

**Figure 6:**
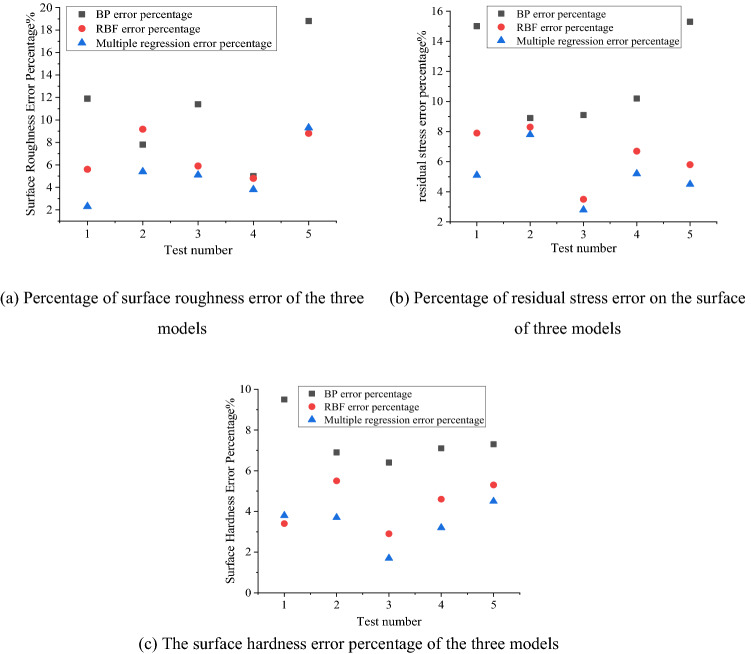
The surface performance error percentage of the three models.

Comparing the prediction model of ultrasonic rolling extrusion surface layer performance established by BP neural network, RBF neural network and multiple regression method, the predicted values of surface roughness, surface residual stress and surface hardness in Tables 5, 7 and 8 were extracted, using origin The software draws a comparison chart of the surface performance test values and predicted values of the three prediction models, as shown in Fig. 5a–c. The error percentages of the three established surface performance prediction models are shown in Fig. 6a–c.

From Fig. 5a–c, it can be seen that in the prediction model of surface performance established by using BP neural network, RBF neural network and multiple regression method, the prediction value accuracy of the mathematical model established by multiple regression is higher than that of BP neural network and RBF neural network prediction model. The accuracy is good, and the prediction point is the closest to the measured point, so the multiple regression method is the best prediction, the RBF neural network is the second, and the BP neural network is the worst.

From Fig. 6a–c, it can be seen that the surface performance prediction model established by the BP neural network has the largest error. The average error percentages between the predicted and experimental values of the surface roughness, surface residual stress and hardness of the RBF neural network are 5.64%, 6.44% and 4.34%, respectively. The average error percentages of surface roughness, surface residual stress and hardness established by multiple regression are 5.18%, 5.08% and 3.38%, respectively. Therefore, the mathematical model of the ultrasonic rolling extrusion surface performance established by the multiple regression method is more accurate and effective, and provides a theoretical model for the subsequent SPEA2SDE optimization.

## Analysis of the multi-objective optimization algorithm

Different from the single objective problem, the optimal solution of the multi-objective problem often appears in the form of a solution set. There is no corresponding optimal solution for all the objective function solution sets but can only coordinate and compromise in all the objective functions so that all the objectives can meet the processing requirements as much as possible^15^. Since Srinivas and DEB proposed a non-dominated sorting genetic algorithm, there have been many excellent multi-objective optimization algorithms, such as NSGA II, MOPSO, AMOSA and SPEA2. However, with the increase of multi-objective optimization function, these traditional multi-objective intelligent algorithms have great difficulties in solving multi-objective problems in three or higher dimensions^16^. To solve the problems of uneven distribution and large jumping of the solution set in the unified multi-objective algorithm, a combination algorithm of SPEA2 and the shift density estimation strategy (SPEA2SDE) is introduced.

### Algorithm evaluation index and performance test

To test the performance of SPEA2SDE, NSGA II and SPEA2, three objective optimization functions are selected, and the test functions are calculated by the SPEA2SDE algorithm, SPEA2 algorithm and NSGA II algorithm to analyse the performance of the three algorithms in the same test function set.

#### Algorithm evaluation index

The quality of the function values should not only be compared. In the experimental comparison of the SPEA2SDE algorithm with the SPEA2 and NSGA II algorithms, in addition to using the test function set, we also need some performance indicators to make a quantitative comparison of the three algorithms.

1. Generational distance
  The generation distance is an important index to measure the convergence of multi-objective problems^17^^.^ It mainly describes the boundary proximity between the Pareto front solution set and the real Pareto optimal solution set:
  8 $$ GD(P,P^{*} ) = \frac{{\sqrt {\sum\nolimits_{x \in P} {d(x,P^{*} )} } }}{\left| P \right|} $$
  where $d(x,P^{*} )$ is the Euclidean distance between the non-dominated solution set X and the solution on the nearest real Pareto front. If the Euclidean distance is smaller, GD will be smaller; that is, the non-dominated solution set is closer to the real Pareto front. Therefore, the smaller the value of GD is, the better the convergence of the algorithm.
2. Reverse generation distance
  The reverse generation distance is an index to evaluate the comprehensive performance of the algorithm. The reverse generation distance is used to express the sum of the distances between the real Pareto front and the Pareto front obtained by the algorithm. The IGD formula is as follows:
  9 $$ IGD(P^{*} ,P) = \frac{{\sum\nolimits_{{x \in P^{*} }} {d(x,P)} }}{{\left| {P^{*} } \right|}} $$
  where $d(x,P)$ is the distance between the ideal Pareto front solution and the algorithm's non-dominated solution. The smaller the value of *IGD* is, the better the distribution and convergence of the algorithm's solution set.
3. Super-volume
  The super-volume HV index is used to express the coverage of the Pareto optimal solution set in a certain region. The excess volume index formula is as follows:
  10 $$ HV = VOL([Q_{1} ,S_{1} ] \times \cdots \times [Q_{n} ,S_{n} ]) $$
  In the formula, VOL is the Lebesgue measure, and the larger the super-volume value is, the better the front-end point of Pareto covers the PF surface, so the larger the HV value is, the better the algorithm is.

#### Test function

Two high-dimensional DTLZ test function sets are selected to simulate and analyse the performance of three multi-objective optimization algorithms^18^. The test function set is shown in Table 9.

**Table 9:** Test function set.

Test function	Mathematical definition	Decision space
DTLZ1	$$\begin{aligned} & \min f_{1} (u) = 1/2u_{1} u_{2} (1 + s(u)) \hfill \\ & \min f_{2} (u) = 1/2u_{1} (1 - u_{2} )(1 + s(u)) \hfill \\ & \min f_{3} (u) = 1/2(1 - u_{1} )(1 + s(u)) \hfill \\ & s(u) = 100\left[ {\left| u \right| + \sum\limits_{t = 3}^{n} {(u_{t} - 0.5)^{2} - \cos (20\pi (u_{t} - 0.5))} } \right] \hfill \\ \end{aligned}$$	$n = 12$ $0 \le x_{t} \le 1$
DTLZ7	$$\begin{aligned} & \min f_{1} (u) = u_{1} \hfill \\ & \min f_{2} (u) = u_{2} \hfill \\ & \min f_{3} (u) = (1 + s(u))/k(f_{1} ,f_{2} ,s) \hfill \\ & s(u) = 1 + 9\sum\limits_{t = 3}^{n} {u_{t} } \hfill \\ & k(f_{1} ,f_{2} ,s) = 3 - \sum\limits_{t = 1}^{2} {(f_{1} /1 + s(u))} (1 + \sin (3\pi f_{1} )) \hfill \\ \end{aligned}$$	$n = 12$ $0 \le x_{t} \le 1$

#### Algorithm performance test

The maximum evolution algebra of the dtlz test function set is 10,000. To ensure the reliability and accuracy of the Pareto solution set obtained from the test function set, the two test function sets are run 30 times to calculate the mean value. The generation distance, anti-generation distance and super-volume test data obtained are shown in Table 10.

**Table 10:** Test data of the test function.

Evaluating indicator	Algorithm	DTLZ1	DTLZ7
GD mean	NSGAII	6.2235e−1	3.4985e−3
	SPEA2	3.1984e−1	4.8013e−3
	SPEA2SDE	1.6562e−1	6.6672e−4
IGD mean	NSGAII	4.4419e+0	8.2667e−2
	SPEA2	2.3473e+0	6.3690e−2
	SPEA2SDE	1.2894e+0	5.8635e−2
HV mean	NSGAII	8.8413e−2	2.6299e−1
	SPEA2	8.0825e−1	2.7236e−1
	SPEA2SDE	8.3249e−1	2.7731e−1

Table 10 shows that the SPEA2SDE algorithm is superior to the SPEA2 and NSGA II algorithms in terms of generation distance, anti-generation distance and super volume calculation in solving multi-dimensional complex multi-objective problems, which shows that SPEA2SDE has better comprehensive performance.

### Pareto front graph of the optimization algorithm

**Figure 7:**
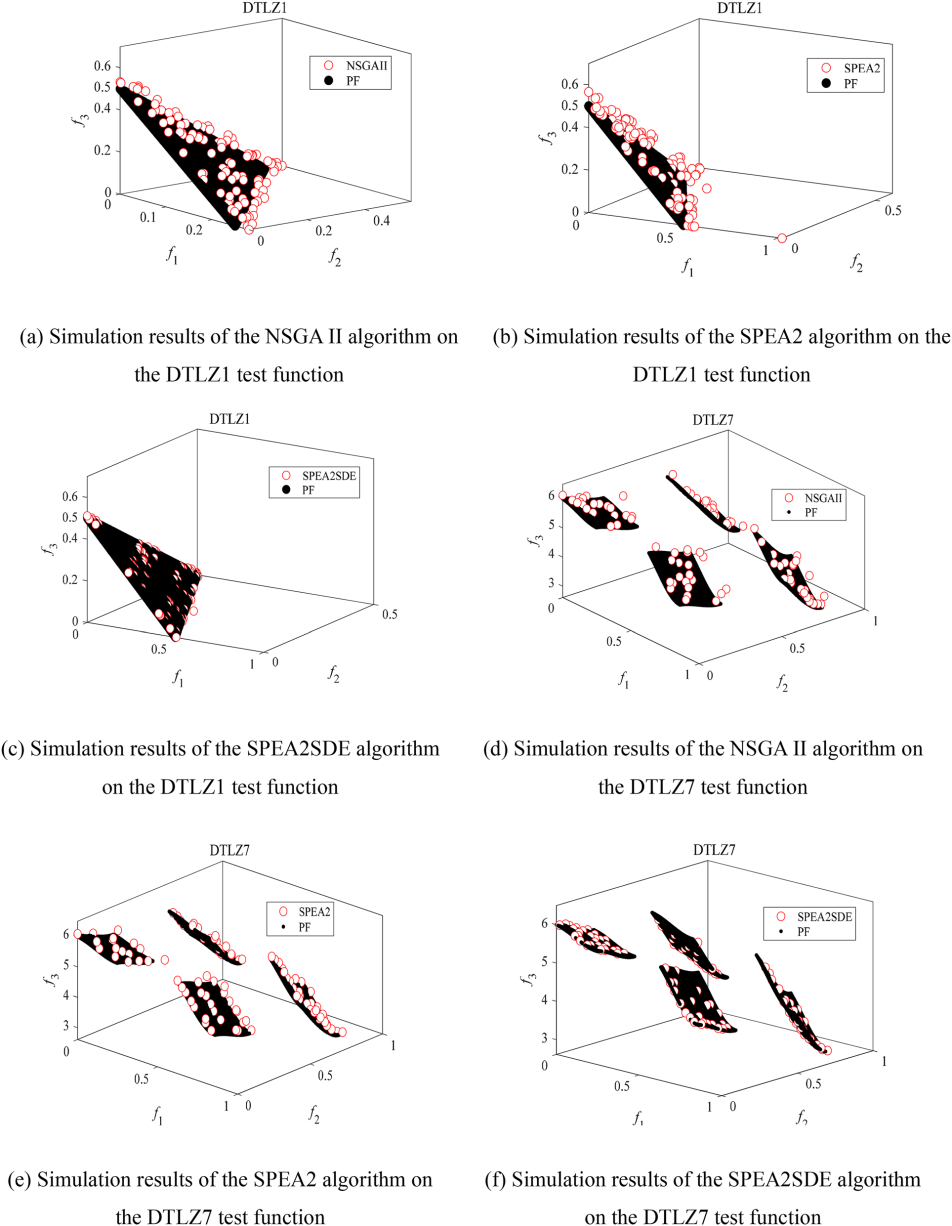
Simulation comparison of three algorithms on DTLZ1, DTLZ7 test function.

To intuitively compare the distribution and convergence of the three algorithms on a real pf surface, the Pareto optimal solution sets of the NSGA II, SPEA2 and SPEA2SDE algorithms in the dtlz series of the three-dimensional test function set are drawn in Fig. 7a–f.

As seen from the figure, the NSGA II algorithm has poor distribution and convergence in 3D optimization, and its solution set tends to be far away from the real pf surface. The SPEA2 algorithm is better than the NSGA II algorithm in three-dimensional multi-objective optimization, but some individual solutions are scattered, which indicates that the algorithm has poor convergence in solving high-dimensional problems. The approximate solution set obtained by the SPEA2SDE algorithm can cover the real pf surface evenly, and the distribution of the solution set is better than that of the other two algorithms. The above results show that the performance of the improved SPEA2 algorithm is improved, especially in the aspect of high-dimensional multi-objective optimization problems, which proves the effectiveness of the SPEA2SDE algorithm and provides a powerful guarantee for the optimization of ultrasonic roll extrusion process parameters.

## Multi-objective optimization of ultrasonic roll extrusion based on the SPEA2SDE algorithm

According to the production requirements of ultrasonic roll extrusion, the objective function formula (5) of surface roughness should be minimized, and the surface hardness and residual stress should be maximized. To obtain the minimum value of the three objective functions, a negative sign should be added before the objective function formula (7) of hardness. Because the residual stress and compressive stress are negative, the objective function formula (6) should be minimized. According to the results of the orthogonal test, the upper and lower limits of each process parameter of ultrasonic roll extrusion are set. The objective function of the surface properties and the constraint conditions of the process parameters are shown in Eq. (11).

11 $$ \left\{ \begin{aligned} & \min R_{a} \left( {n,f,A,F} \right) \hfill \\ & \min \sigma \left( {n,f,A,F} \right) \hfill \\ & \min - HV\left( {n,f,A,F} \right) \hfill \\ & 150 \le n \le 630 \hfill \\ & 15 \le f \le 59 \hfill \\ & 200 \le F \le 600 \hfill \\ & 5 \le A \le 29 \hfill \\ \end{aligned} \right. $$

**Figure 8:**
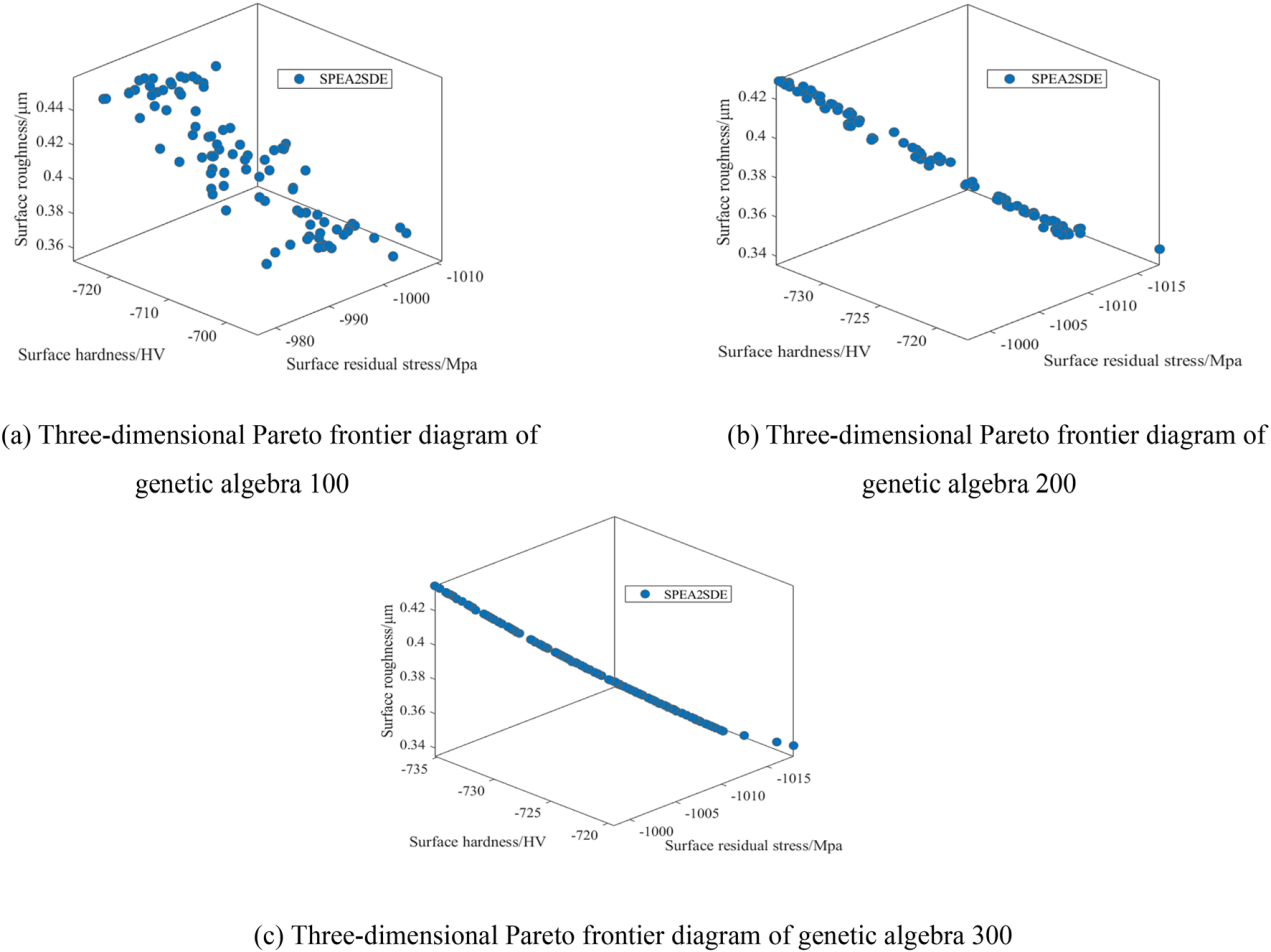
Three-dimensional Pareto frontier diagram of different genetic algebras.

To reduce the surface roughness and improve the surface residual stress and surface hardness, the multi-objective optimization model of the ultrasonic roll extrusion process parameters was optimized by the SPEA2SDE algorithm with the spindle speed, feed speed, ultrasonic amplitude and static pressure as optimization variables. The evolution algebra was 100, 200, and 300, the crossover factor was 0.90, and the mutation probability was 0.3. The Pareto frontier graphs of three genetic algebras are obtained, as shown in Fig. 8a–c. The top 10 of 120 sets of Pareto optimal solution sets with 300 generations of evolution algebra are listed in Table 11.

**Table 11:** Pareto frontier solution of the SPEA2SDE algorithm.

Serial number	*n*	*f*	*F*	*A*	$R_{a}$	$\sigma$	$HV$
1	193	20	561	26	0.354	− 1018	− 707
2	196	20	562	26	0.337	− 1017	− 725
3	200	23	559	27	0.364	− 1011	− 728
4	200	20	560	28	0.338	− 1016	− 725
5	200	18	561	26	0.349	− 1018	− 711
6	201	20	558	26	0.389	− 1006	− 730
7	201	20	560	28	0.366	− 1010	− 728
8	201	23	563	26	0.417	− 1001	− 733
9	199	25	562	26	0.420	− 999	− 734
10	197	20	560	26	0.352	− 1018	− 709

As seen from Fig. 8a–c, when the evolution algebra is 100 generations, the convergence of the Pareto front solution is poor, and the distribution is uneven. When the evolution algebra is 200 generations, the convergence of the Pareto front solution is still poor. When the population evolves to 300 generations, good optimization results are obtained. In conclusion, when the evolution algebra is 300, the Pareto solution set is more stable, accurate and evenly distributed, which proves that the algorithm has better robustness and convergence. Because a larger algebra will affect the operation speed of the algorithm, the evolutionary algebra of this optimization is selected as 300.

## Test verification

**Figure 9:**
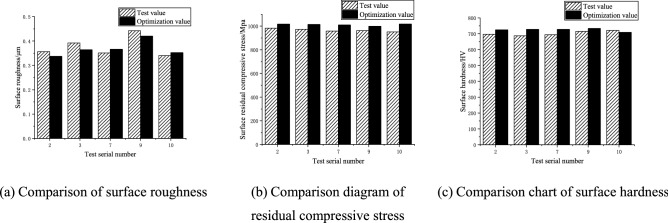
Optimized and experimental values of the surface layer performance of ultrasonic roll extrusion.

To verify the effectiveness of the SPEA2SDE algorithm in optimizing ultrasonic roll extrusion process parameters, five groups of optimization results were randomly selected for experimental verification, and the optimization results of groups 2, 3, 7, 9 and 10 in Table 11 were selected. Because the hardness value was positive, the negative sign was removed, and the results are shown in Table 12. To compare the optimized value and the experimental value more intuitively, a histogram of the optimized value and the experimental value of the surface properties of 42CrMo steel is drawn, as shown in Fig. 9.

**Table 12:** Results of the optimized and experimental values of the surface layer performance by ultrasonic rolling.

$R_{a}$/μm			$\sigma$/MPa			Hardness/HV		
Actual value	Optimization value	Error (%)	Actual value	Optimization value	Error (%)	Actual value	Optimization value	Error (%)
0.356	0.337	5.3	− 983	− 1017	3.4	697	725	4.0
0.392	0.364	7.1	− 973	− 1011	3.9	687	728	5.9
0.351	0.366	4.2	− 958	− 1010	5.4	694	728	4.9
0.442	0.420	5.0	− 962	− 999	3.8	715	734	2.6
0.340	0.352	3.5	− 951	− 1018	7.0	721	709	1.7

Table 12 shows that the average error percentages of the actual and optimized values of surface roughness, surface residual stress and surface hardness are 5.02%, 4.7% and 3.82%, respectively. In the optimization of surface roughness, the error percentage of the third group of data is the largest, which is 7.1%. In the optimization of residual stress, the error percentage of the tenth group of data is the largest, which is 7.0%. In the optimization of surface hardness, the error percentage of the third group is the largest, which is 5.9%. In general, the average error between the experimental value and the optimized value of 42CrMo steel surface properties is controlled within 10%, which shows that the SPEA2SDE algorithm has high accuracy and good reliability in solving the multi-objective optimization model of ultrasonic roll extrusion, which can be used to guide actual production and processing.

## Conclusions

To obtain better surface properties of 42CrMo steel and balance the relationship among surface roughness, surface residual stress and hardness, the ultrasonic roll extrusion process parameters were optimized by orthogonal testing, regression analysis and the SPEA2SDE algorithm. The results are summarized as follows:

1. Based on the orthogonal experiment, a multiple regression model of surface roughness, surface residual stress and hardness was established by the multiple regression method, and the significance of the model was tested by variance analysis. The negative correlation coefficients of the model and HV are greater than 0.99, which indicates that the model has a good fit and can be used as the objective function of the subsequent multi-objective optimization model.
2. The SPEA2SDE algorithm is used to optimize the process parameters of ultrasonic roll extrusion, and some of the solutions are verified by experiments. The results show that the average error percentages of the actual value and the optimized value of surface roughness, surface residual stress and surface hardness are 5.02%, 4.7% and 3.82%, and the average error of the surface performance test value and the predicted value is controlled within 10%, which proves that the algorithm has good robustness and high optimization accuracy.
3. The SPEA2SDE optimization algorithm can obtain a good Pareto optimization front end with a faster convergence speed, which provides an effective method for selecting an optimization scheme in actual production and processing and greatly improves production efficiency.

## Data Availability

The data that support the findings of this study are available on request from the corresponding author. The data are not publicly available due to privacy or ethical restrictions.
